# Modulating Transthyretin Fibril Stability with D‑Retro-Inverso Peptides

**DOI:** 10.1021/acs.jcim.6c02525

**Published:** 2026-10-27

**Authors:** Lucy M. Coleman, Ulrich H. E. Hansmann

**Affiliations:** Department of Chemistry & Biochemistry, 6187 University of Oklahoma, Norman, Oklahoma 73019, United States

## Abstract

A major cause of heart failure in elderly patients is the deposition of transthyretin (TTR) fibrils. Using molecular dynamics simulations, we explore how the stability of TTR fibrils can be modulated by D-retro-inverso (DRI) peptides, built from D-amino acids with the sequence of the parent peptide switched. We screened a library of peptide candidates generated from aggregation-prone regions in the TTR sequence, including peptides from WALTZ-DB 2.0 database of amyloid peptides, and selected three peptides based on their potential to disrupt interactions in a structurally flexible region of the fibril. Peptides were docked to the surface of the TTR fibril using HADDOCK 2.4, followed by all-atom molecular dynamics simulations to evaluate peptide–fibril interactions and their effects on fibril structure. We finally describe a potential mechanism by which one of these peptides, DRI-K6V, perturbs the structural integrity of TTR fibrils, and identify the segment K80-S100 as a potential target for modulating TTR fibril stability. Our results may open the way to the design of peptide drugs targeting established TTR amyloidosis.

## Graphical abstract

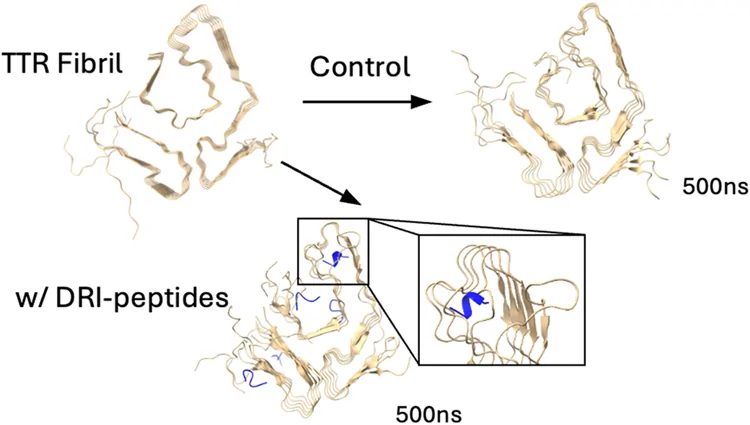

## Introduction

The extracellular protein transthyretin (TTR) assembles as a tetramer transporting thyroxin and retinol throughout the body. Upon dissociation of the tetramer, the released monomers can partially unfold and aggregate into insoluble fibrils that may damage the heart and nervous system but can also affect the eye, kidney, and the gastrointestinal tract.^1^ Wild-type transthyretin amyloidosis (ATTRwt), where amyloid deposits are found mainly in the heart, is estimated to affect over 300,000 elderly patients worldwide, while the hereditary (i.e., mutant-based) form of disease, transthyretin amyloidosis (ATTRv), is seen in about 50,000 patients.^2–4^ Early detection of the disease is rare, and the mean survival times in untreated patients are about 2.5–3.6 years after diagnosis.^5–8^ Current treatment options are limited to management and preventing aggravation of symptoms. Heart transplants can replace diseased hearts in patients with ATTRwt or severe ATTRv. Liver transplants can remove the source of mutant TTR in patients with ATTRv,^3^ and RNAi treatments, patisiran and vutrisiran, can inhibit expression of TTR.^9–12^ Other U.S. Food and Drug Administration (FDA)-approved drugs, such as tafamadis,^13^ diflusinal,^14,15^ and acoramidis,^16,17^ prevent aggregation into fibrils by stabilizing the TTR tetramer. Common to all these approaches is that they reduce the progression of fibril formation but cannot eliminate the existing disease-symptoms causing amyloid deposits. Hence, as existing therapies have limited effectiveness during the late stages of the disease, there is a need for exploring avenues to inhibit or even reverse TTR fibril formation.

One possibility is the use of small peptides that can bind to TTR fibrils, destabilizing and dissolving them. In the context of other amyloid-forming human proteins, short peptides have been shown to inhibit aggregation by acting as β-sheet breakers.^18–22^ Well studied are peptides that are built from the proteolysis-resistant D-amino acids, as they avoid short *in vivo* lifetimes that would limit their therapeutic use.^23^ One possibility is the use of D-retro-inverso (DRI)-peptides, where reverting the amino acid sequence and flipping the chirality of amino acids from l-enantiomers to d-enantiomers leads to molecules that resemble the parent peptide and often maintain their biological activity while avoiding proteolysis.^24^ For instance, we showed in previous work that *in silico*, a five-residue peptide, RSFFS, and its DRI form, DRI-R5S, destabilize serum amyloid A (SAA) fibrils.^25^ A subsequent *in vivo* study demonstrated that in a mice model treatment with DRI-R5S after myocardial infarction decreases formation of SAA fibrils, resulting in improved heart function and recovery.^26^ Motivated by these previous results, we propose a computational framework using virtual screening to identify and evaluate DRI peptides for their ability to destabilize TTR amyloid fibrils, and molecular dynamics (MD) simulations to identify their mechanism of action. While previous studies focused on short peptides that inhibit fibril formation and growth by interacting with the TTR monomer,^27^ binding to soluble oligomers or capping fibril ends,^28–32^ we are interested in peptides capable of perturbing mature fibrils, which could inform the development of therapeutic strategies for targeting established amyloid deposits.

We remark that we did not address the specificity of our peptide candidates, i.e., their binding to competing proteins in the heart or other organs. This is because we are mainly interested in understanding the mechanism by which peptides can destabilize TTR fibrils, a question that is difficult to access with current experimental approaches. To our knowledge, there are only two (transgenic double-humanized) mouse strains (hV/hV:hR/hR and hV/hM:hR/hR) where TTR amyloids are observed in the heart and that therefore could serve as a mouse model.^33^ However, amyloid deposition is seen only at 24 months of age, i.e., in mice of advanced age, and appears to be very sensitive to environmental conditions. This makes *in vivo* experiments on TTR amyloidosis difficult and costly. Hence, a detailed knowledge of the mechanism by which peptides can dissolve the TTR fibril is needed for optimizing the potency and selectivity of potential drug candidates prior to experimental validation. For this purpose, we identify in the present paper suitable peptides and try to understand the mechanism by which they cause destabilization of the TTR fibril. Using high-throughput docking and Rosetta energy analysis to screen a library of candidate peptides, we selected final candidates based on predicted engagement with dynamically vulnerable regions of the fibril as identified by MD simulations of an experimentally resolved TTR fibril model. MD simulations of the top candidates interacting with TTR fibrils are used to validate promising binders and allow us to identify the mechanism by which one of these peptides, DRI-K6V, promotes structural destabilization of TTR fibrils.

## Methods

### Generation of Fibril Model

The TTR fibril model was generated using the cryo-EM-derived structure of a patient-derived wild-type TTR fibril at a resolution of 2.78 Å as deposited in the Protein Data Bank (PDB) with PDB ID: 8ADE.^34^ Several other studies have also posed models of TTR fibrils, all revealing a common fold with several unresolved regions: the first ten residues of the N-terminus (residues G1-C10), the last four residues of the C-terminus (residues N124-E127), and a 21-residue segment in the middle of the protein (residues A36-H56).^35–37^ For our simulations, we added the missing N- and C-terminal regions using the MODELLER software for de novo loop modeling.^38^ On the other hand, the unresolved segment (residues A36-H56) was not added under the hypothesis that it is proteolytically cleaved during fibril formation, consistent with mass spectrometry data showing that most fibril chain fragments begin or end within this region.^36,37,39^ Hence, our fibril model of five TTR chains consists of two “proto fibrils” made of fragments G1-K35 and G57-E127; see Figure 1a.

**Figure 1:**
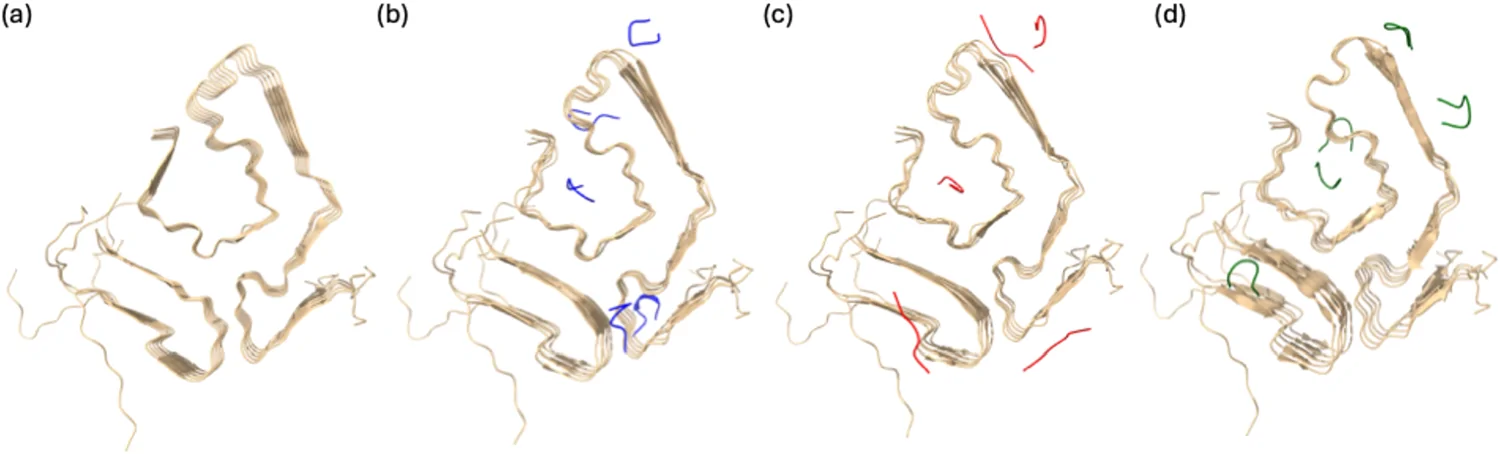
Start conformations of simulations of (a) a sole TTR fibril model (our control), and in the presence of three DRI peptides: (b) DRI-K6V (in blue), (c) DRI-W6I (in red), and (d) DRI-W6Y (in green).

### Peptide Library Generation

Amyloidogenic regions of the TTR protein sequence were identified using web-based software TANGO,^40^ AGGRESCAN,^41^ ZipperDB,^42^ WALTZ^43^ and PASTA.^44^ We focus on hexapeptides based on extensive studies on six-residue amyloidogenic peptides, which is around the length of a typical β-strand.^45,46^ All unique six-residue sequences were extracted from amyloidogenic regions of TTR using a sliding window approach. The library was generated through mutational analysis of the six-residue amyloidogenic sequences, where amino acids were replaced only with those belonging to the same group as defined by the chemical properties of their side chains (Table ST1). We considered only one-point and two-point mutated peptides, i.e., such that they differ from the parent sequence by only one or two residues. In this way, we generated a total of 9,297 peptides.

The solubility of each of these peptides was evaluated by the Kyte-Doolittle Hydropathy^47^ scale, and the β-strandness of each peptide was screened by the Chou and Fasman^48^ parameters. Peptides with moderate hydrophobicity (0.8–1.8) and moderate overall β-strand propensity (1.0–1.2) were considered for further screening to ensure structural stability for target binding, reduced tendency to aggregate, and solubility, all characteristics seen in inhibitory peptides targeting other amyloid diseases.^49,50^ Next, the peptides were screened for amyloidogenicity by AmyloGram,^51^ AGGRESCAN,^41^ and ZipperDB.^42^ Sequences identified to be amyloidogenic were used for molecular docking, generating a final library of 1,134 peptides. In addition to our library of peptides based on amyloidogenic sequences of the TTR protein, we also considered the WALTZ-DB 2.0 database^46^ containing 1404 unique hexapeptides with amyloid-forming potential.

All considered peptides were converted to their DRI form by reversing the sequence, generating structures with PeptideBuilder,^52^ and converting each amino acid from the l-enantiomer to the d-enantiomer by swapping the positions of the β-carbon and α-hydrogen. The resulting peptides were energy-minimized in implicit solvent using SANDER from AmberTools with the Generalized Born implicit solvent model.^53^

### Peptide Library Screening

For an initial high-throughput docking, we used AutoDock Vina version 1.2.5 with a grid search area of 81.810 Å x 95.640 Å x 58.440 Å and center coordinates of 60.565, 60.070, 60.560 Å.^54^ Peptides were categorized as one-point mutations, two-point mutations, or WALTZ peptides. The top 100 scoring peptides from each category were selected for further analysis.

Based on the AutoDock Vina binding poses, we observed that the peptides preferred docking to the central polar cavity. Additionally, our 400 K molecular dynamics simulations of the fibril identified residues K80-S100 as an early region to destabilize. These residues are part of the top β loop and include side chains (e.g., Phe, Leu, Trp, Tyr, Ile) forming fibril-stabilizing interchain contacts. Therefore, we refined our search to peptides that could bind to the central cavity and perturb this hotspot region. Starting from the top 100 AutoDock Vina binding poses of each category (one-point mutations, two-point mutations, and WALTZ peptides), all complexes were subjected to constrained FastRelax in Rosetta, and the lowest-energy model was retained. Binding energetics were evaluated using Rosetta InterfaceAnalyzer to compute the binding free energy (dG_separated), buried surface area (dSASA_int), interfacial hydrogen bonds (hbonds_int), and buried polar atoms (delta_unsatHbonds) (Table ST2). Binding free energy estimates Δ*G* are reported in Rosetta Energy Units (REU). Per-residue energy decomposition was performed for the top 10 candidates, extracting both one-body and pairwise terms.

Candidates were analyzed by their Rosetta binding free energy estimate, contacts with hotspot residues K80-S100 per chain (weighted by per-residue fluctuation from the 400 K control simulation; see discussion in the Results and Discussion section), hydrophobicity, and per-residue energy decomposition at the hotspot region. The five candidates showing the strongest perturbation of residues K80-S100 (DRI-KALGIV, DRI-YTIGAL, DRI-WTFNLY, DRI-VALHGF, DRI-WKALLI) were selected for independent validation with HADDOCK 2.4 ^55^ using residues of the fibril cavity as active residues. The resulting structures clustered by root-mean-square deviation (RMSD) between each other, and the lowest-energy structure from the top-ranked cluster was selected for downstream analysis. HADDOCK poses were relaxed and scored using the same Rosetta protocol. Final candidates were selected based on per-residue energetics at the hotspot region across both AutoDock-Rosetta and HADDOCK-Rosetta analyses. The candidates were also prioritized for potential mechanistic diversity, as their per-residue energy patterns suggested distinct modes of cavity engagement. Three peptides were selected as final candidates for MD simulation—DRI-KALGIV (DRI-K6 V), DRI-WKALLI (DRI-W6I), and DRI-WTFNLY (DRI-W6Y). DRI-K6 V and DRI-W6I are one-point mutations generated from amyloidogenic sequences of TTR, and DRI-W6Y comes from the WALTZ-DB 2.0 database.

DRI-K6V and DRI-W6I are derived from adjacent segments of TTR ^80^KALGIS^85^ and ^79^WKALGI^84^, respectively. In the native TTR protein, the ^80^KALGIS^85^ segment spans the C-terminal end of the EF helix (T75-L82) and the start of the EF loop (G83-E89), a region that stabilizes both the strong and weak dimer interfaces in the homotetramer but becomes conformationally dynamic at acidic pH, with hereditary mutations in this region accelerating aggregation.^56,57^ Additionally, this segment has been identified through crystallography as an amyloidogenic core of TTR, forming an in-register steric zipper as in the fibril spine.^58^ DRI-K6V preserves the native Leu82/Ile84 hydrophobic pair that forms the steric zipper, with the substitution of Ser to Val adding a branched aliphatic side chain that increases β-strand propensity and hydrophobicity.^58^ DRI-W6I has a Trp residue capable of both hydrophobic packing and π-stacking and replaces the native Gly with Leu, increasing in this way the overall hydrophobic surface area. DRI-W6Y, an amyloidogenic peptide from the WALTZ-DB database, is derived from residues 140–145 of KCNIP1, a calcium-binding EF-hand protein that regulates Kv4-type voltage-gated potassium channels.^46^ It contains large aromatic residues (Trp, Phe, Try) which support both hydrophobic contacts and π-stacking interactions.

We remark that our three peptide candidates form, upon docking, compact conformations that permit insertion into the polar cavity of the TTR fibril, potentially disrupting the interstrand hydrogen bonding networks, electrostatic interactions, and hydrophobic packing that maintain the fibril, see Figure 1b–d. On the other hand, the TabF2 and TabH2 TTR-targeting peptides previously developed by the Eisenberg group^59^ adopt upon docking an extended conformation stretched on the surface perpendicular to the fibril axis; see Figure SF1. As this arrangement effectively prevents further growth of fibrils but does not destabilize existing fibrils, we did not consider TabF2 and TabH2 in our study.

### General Simulation Protocol

To generate the initial structures for the molecular dynamics simulations, all starting structures were converted to GROMACS format using pdb2gmx, and hydrogens were added to the structures. The N- and C-terminals of the fibril were left charged, and the ends of the resolved fragments were also left charged as if they were cleaved. The N- and C-termini of the peptides were capped with an NH_2_ and CONH_2_ group, respectively, in order to counteract electrostatic repulsion between the peptide’s terminal groups. The fibril contains five chains, and the peptides were docked to the surface of the fibril in a 1:1 ratio using HADDOCK 2.4 software.^55^ For each system, the first peptide was docked to the central polar cavity, while the remaining four were docked using random surface-exposed patches. The resulting simulation boxes for DRI-K6V, DRI-W6I, and DRI-W6Y had volumes of 1897.41, 2202.07, and 2005.14 nm^3^, corresponding to effective concentrations of approximately 3.8, 4.4, and 4.1 mM for the five peptide copies, respectively. This concentration is substantially higher than concentrations that would be expected in a pharmacological setting, a common problem in molecular simulations. However, the purpose of the simulations was to characterize the molecular interactions and binding behavior of individual peptide molecules at the fibril surface. The use of multiple peptide copies allowed us to sample interactions at multiple surface-exposed sites within the same fibril model. Sketches of the so-generated start conformations are shown in Figure 1, and the corresponding atomic coordinates are given as Supporting Information (TTR-fibril-coordinates.zip). Note that the peptides were free to move or disengage from the fibril throughout the simulation.

Depending on the availability of computational resources, simulations utilized either GROMACS 2022 or 2020.4 software:^60^ GROMACS 2022 for the control and DRI-K6V systems and GROMACS 2020.4 for DRI-W6I and DRI-W6Y systems. All simulations relied on the CHARMM36m all-atom force field^61^ and TIP3P explicit water model,^62^ and identical simulation parameters and analysis protocols were applied across all runs. The fibril system was placed in a cubic box with a minimum distance of 15 Å between the solute and the edge of the box and periodic boundary conditions. The system was solvated, and counterions were added to neutralize the system in accordance with the particle-mesh Ewald method. Na^+^ and Cl^–^ ions were added at a physiological concentration of 150 mM. To ensure that the starting configuration is a local minimum, we minimized each system for up to 50,000 steps using the steepest descent method, followed first by 200 ps of molecular dynamics at constant volume and temperature of 310 K, and afterward 200 ps at a constant pressure of 1 atm. Heavy atoms were constrained with a force constant of 1000 kJ/mol•nm^2^. Production runs were performed at a constant temperature of 310 K and constant pressure of 1 atm. We considered three independent trajectories of 500 ns each to capture global structural changes in the presence of the peptides.

The temperature of the simulations was maintained using a velocity-rescale thermostat^63^ with a coupling constant of 0.1 ps, and pressure was controlled with the Parrinello–Rahman barostat^64^ with a relaxation time of 2 ps. Equations of motion were integrated with a 2 fs time step, enabled by constraining water geometry with the SETTLE^65^ algorithm and protein bonds containing hydrogen atoms to their equilibrium lengths with the LINCS^66^ algorithm. Periodic boundary conditions were applied to all systems, with long-range electrostatics computed using particle-mesh Ewald with a real-space cutoff of 12 Å and a Fourier grid spacing of 1.6 Å. Short-range van der Waals interactions were truncated at 12 Å with smoothing from 10 Å. Each system was simulated in triplicate with differing initial velocity distributions. The total number of atoms and water molecules from each system, as well as the length of trajectories for each system, is listed in Table 1, and the corresponding atomic coordinates of the start and final conformations are given as Supporting Information (TTR-fibril-coordinates.zip).

**Table 1:** Simulated Systems

system	total atoms	water molecules	individual simulation time (ns)	total simulation time (ns)
control	176,260	56,037	500	1500
DRI-K6V	191,683	61,016	500	1500
DRI-W6I	216,748	69,338	500	1500
DRI-W6Y	197,847	63,039	500	1500

### Trajectory Analysis

MD trajectories were analyzed with GROMACS tools^60^ and visualized using VMD^67^ and UCSF ChimeraX.^68^ GROMACS tools were used to calculate root-mean-square deviation (RMSD), root-mean-square fluctuation (RMSF), radius of gyration (*R*_g_), solvent-accessible surface area (SASA) with a spherical probe of 1.4 Å, and pairwise distances used for contact analysis. All contacts are defined at a cutoff of 4.5 Å between heavy atoms in a residue pair. Stacking contacts refer to contacts between chains of the fibril, while packing contacts are defined as contacts between the N-terminal fragment (residues G1-K35) and the C-terminal fragment (residues G57-E127).

## Results and Discussion

In order to evaluate the ability of our three peptides to destabilize and disrupt the TTR fibril, we compare molecular dynamics simulations where the peptides interact with the fibrils with those where the peptides are absent. The evolution of the fibril conformations was followed for each system in three independent trajectories over 500 ns at a physiological temperature of 310 K and a standard pressure of 1 atm. The atomic coordinates of the final conformations of all trajectories are also given as Supporting Information in the compressed folder TTR-fibril-coordinates.zip. To quantify structural changes, we have calculated as a function of time the root-mean-square deviation (RMSD) over all heavy atoms with respect to the starting conformation. This quantity changes little with time in the control simulations and in all cases approaches, within 300 ns, a plateau; see Figure 2. Hence, when calculating averages, we considered only the last 200 ns of our trajectories. Note that these averages do not indicate an equilibrium state but rather that quantities change little when going from an interval of, say, 300–400 ns to 400–500 ns (data not shown), allowing in this way to compare the various systems in a simple way. A visual inspection of the final conformations (see the insets to Figure 2) shows little changes, confirming the known stability of the TTR fibril, with structural changes mainly in the polar cavity of residues G57-G83. This is not surprising as residues A36-H56 are assumed to be proteolytically cleaved during fibril formation; i.e., our fibril model consists of two proto fibrils, and we expect flexibility of the end residues K35 and G57.

**Figure 2:**
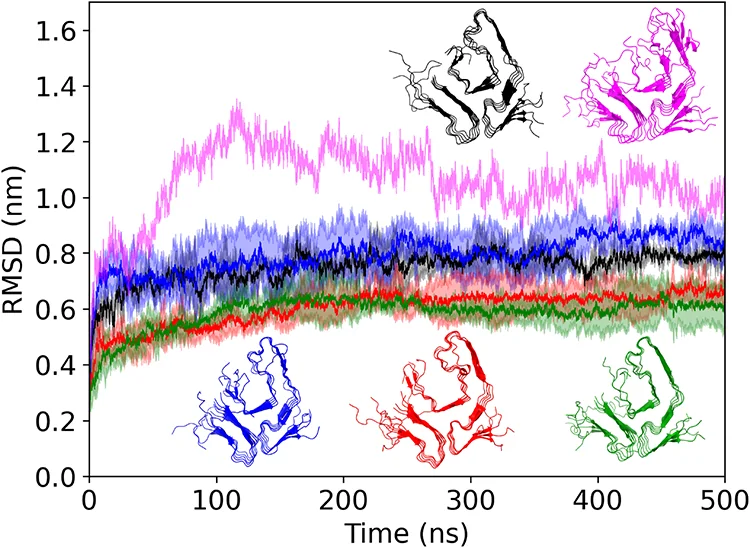
Root-mean-square deviation (RMSD) to the start configuration, calculated over all heavy atoms, as a function of time for the TTR fibril in the presence of DRI-K6V (blue), DRI-W6I (red), DRI-W6Y (green), and the control (black), shown as averages over three trajectories. We draw the RMSD of the individual trajectory for the additional control simulation at the unphysiological high temperature of 400 K in magenta. The final conformations of all five systems are drawn using the same color coding.

This increased flexibility for residues G57 to G83 is also seen in Figure 3, where we show the residue-wise root-mean-square fluctuation (RMSF) calculated over the last 200 ns and averaged over all five chains. However, interestingly, we see in Figure 3 a slightly raised RMSF for the segment of residues K80-S100, corresponding to the loop connecting strand G (residues K76-K80) of the polar cavity with strand H (residues V93-N98), indicating flexibility in the spatial arrangement between the cavity and the adjacent strand H. However, this signal is weak, and to obtain a clearer picture, we added another control simulation at an unphysiologically high temperature of 400 K, where we expect a weakening of the fibril. The RMSD as a function of time and the final conformation of this trajectory are also shown in Figure 2, both indicating a partial dissociation of the TTR fibril, with not only the polar cavity but also strand H distorted. Comparing the residue-wise RMSF of this trajectory with the ones from simulations at the physiological temperature of 310 K, we see now a clear signal for increased flexibility of the K80-S100 segment. This flexibility allows, in the biologically active structure of the TTR protein (which assembles into a homotetramer), hydrogen bonding between the segments of residues A91–A97 on adjacent strands of the four chains in the dimer of dimers. Hence, we hypothesize that this flexibility, while needed in the native structure, could represent a potentially exploitable region for peptide interactions in the fibril. The enhanced response of this region at an elevated temperature was therefore used as a guide for identifying a candidate site at which peptide interactions might perturb the fibril. This hypothesis was a guiding assumption in the search for binding sites for the peptides to the TTR fibrils.

**Figure 3:**
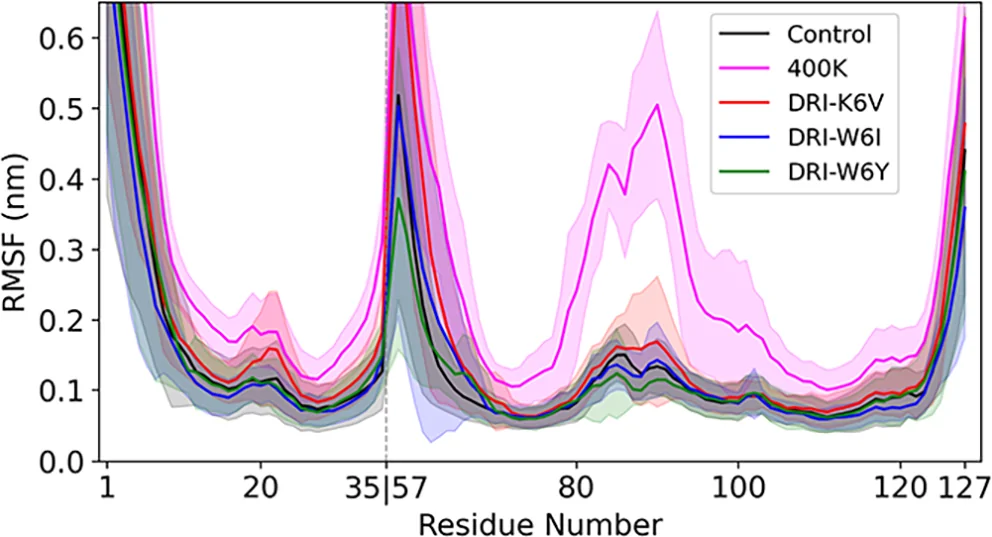
Residue-wise root-mean-square fluctuation (RMSF) calculated for each system over the last 200 ns and averaged over all three trajectories and five chains in the TTR fibril.

Figure 1 shows the starting conformation of three sets of simulations where our DRI peptides, in a one-to-one ratio, are interacting with the TTR fibril. In all three cases are at least some of the peptides binding with the polar cavity in the initial conformation. Although peptides were initially positioned on the fibril surface based on HADDOCK docking, they were not restrained to these configurations. The peptides departed from their initial docking poses and dynamically sampled the fibril surface throughout the simulations. Consequently, the subsequent trajectories were used to characterize peptide–fibril interactions and their effects on fibril structure rather than to evaluate the persistence of the initial docked complexes. We show in Figure 4a–c the probabilities of the three peptides to bind to a specific TTR residue as measured over the last 200 ns and averaged over three independent runs. Corresponding binding probabilities for the peptide residues are shown in Figure 5a–c.

**Figure 4:**
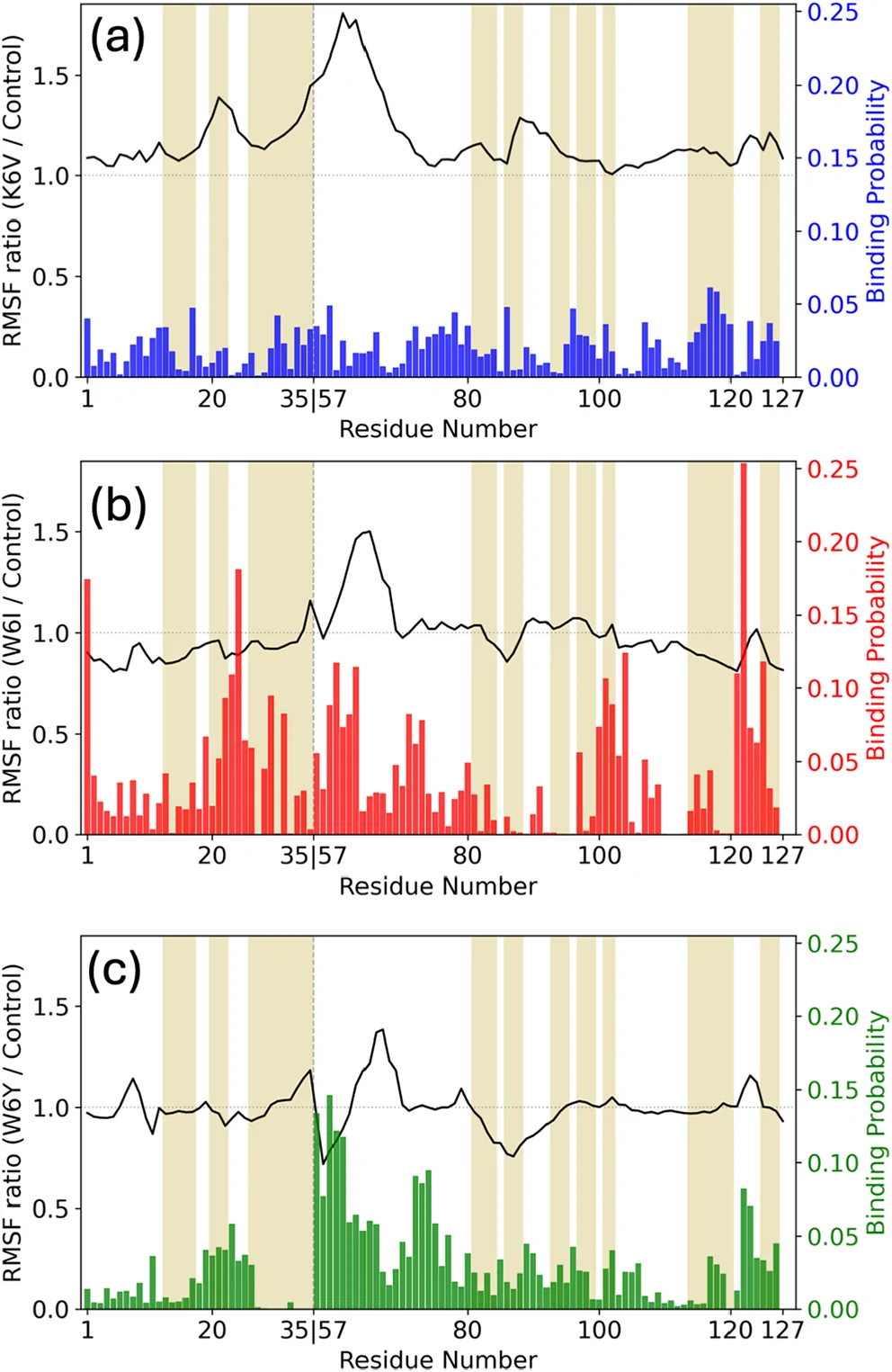
Bar graphs of the per-residue binding probability of DRI peptides to the TTR fibril model for (a) DRI-K6V (blue), (b) DRI-W6I (red), and (c) DRI-W6Y (green). The black line marks the ratio of the residue-wise RMSF measured in such simulations divided by the corresponding value in the control simulation. The β-strand regions in the TTR fibril are shaded in yellow.

**Figure 5:**
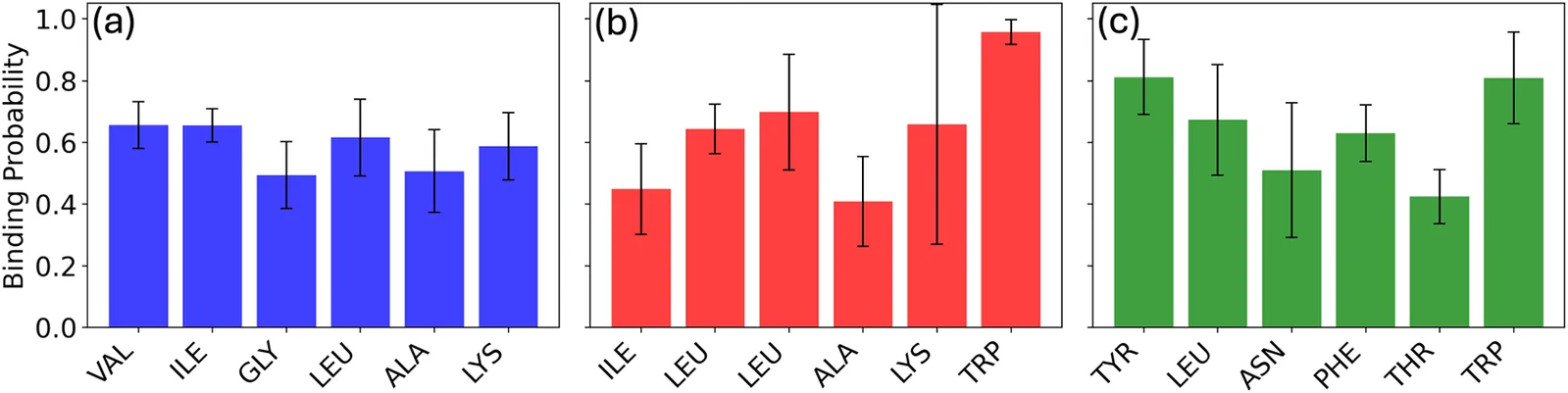
Binding probabilities of the DRI peptide residues binding to the TTR fibril for (a) DRI-K6V, (b) DRI-W6I, and (c) DRI-W6Y.

We first note that in Figure 5a, the residues of DRI-K6V have all similar probabilities to bind to the TTR fibril, while the distribution is more varied for DRI-W6I (Figure 5b) and DRI-W6K (Figure 5c). The two peptides include aromatic residues that have binding frequencies above 90%. Overall, the frequency of DRI-K6V binding with at least one residue to the TTR fibril is 77%, much lower than the corresponding frequencies of DRI-W6I (99%) and DRI-W6Y (95%). The elevated per-residue binding probabilities seen for DRI-W6I and DRI-W6Y are consistent with large aromatic residues that can provide anchoring interactions. Both contain Trp, and DRI-W6Y also contains Phe and Tyr residues which can interact with the TTR fibril surface through π-stacking and extended van der Waals contacts. The more uniform binding probabilities across all DRI-K6V residues suggest a lack of a dominant interaction, where contacts are instead distributed across several weak and nonspecific interactions.

Using the approach described by Bellaiche and Best,^69^ we find that DRI-K6V has a free energy of binding to the fibril of −17 kJ/mol, binding less strongly to the TTR fibril than DRI-W6I (−29.3 kJ/mol) and DRI-W6Y (21.4 kJ/mol). This weaker binding can also be seen in Figure 4a–c, where we show the binding probabilities to the TTR residues. As a rule, they are below 7% for DRI-K6V and usually higher for the other two peptides. The distribution for DRI-K6V (Figure 4a) is also more even than for DRI-W6I (Figure 4b) or DRI-W6Y (Figure 4c), and could indicate that DRI-K6V acts through a diffusion mechanism, which in other contexts has been shown to be effective in disrupting specific molecular interactions.^70^ On the other hand, DRI-W6I and DRI-W6Y would have to act through more localized interactions, as we find here peaks that correspond to preferred binding sites. In the case of DRI-W6I, these correspond to strands C (A25-I26), E (T59-E62), F (E72-I73), I (R104-T106), and L (T118-V122), and for DRI-W6Y to strands E (T59-E62), F (E72-I73), and L (T118-V122). Hence, both peptides have the polar cavity and the adjacent strand I as preferred binding sites, while this is not the case for DRI-K6V. However, we find for none of the three peptides a signal for significant binding to the region of residues K80-S100, which our control simulations marked as critical.

Note that the initial sites and the binding probabilities differ from those seen for the inhibitor candidates TabF2 and TabH2 suggested earlier by the Eisenberg group. These peptides were initially designed to bind to the native TTR F and H strands, believed to be involved in the aggregation process, then further optimized for solubility and to limit self-aggregation by the addition of a four-residue arginine tag and an *N*-methyl group to generate two 16-residue peptides named TabF2 and TabH2.^27,59^ As described by us in the Methods section, these peptides adopt an extended conformation across the fibril surface (Figure SF1), consistent with a surface-capping mechanism. Both *N*-methylation and a positively charged polyarginine tag optimize solubility and electrostatic recognition at fibril termini but appear to constrain conformational flexibility and restrict penetration into the polar cavity. Notably, despite their high potency as fibril growth inhibitors, TabF2 and TabH2 peptides do not destabilize or degrade existing preformed fibrils.^29^ We expect that this is different for our peptides, which clearly interact with the polar cavity and the interior of the fibril; see also the peptide binding probability for the five chains of the TTR fibril model shown in the supplemental Figure SF2.

The RMSD plot in Figure 2 indicates that the effect of this interaction between the peptides and the TTR fibril is, for DRI-K6V, a loosening of the fibril geometry consistent with the assumption that peptides derived from a protein’s own sequence can bind and inhibit its aggregation.^71^ Visual inspection of the final conformation in the inset of Figure 2 and of the residue-wise RMSF in Figure 3 suggests that the locus of the changes is indeed the same as in the high-temperature control simulation, indicating that DRI-K6V exploits the presumed structural weakness of the TTR fibril. Note also that the binding sites correspond to minima in the relative RMSF curves of Figure 4a–c, which show the ratio of the RMSF measured in simulations with the peptide present divided by the RMSF values measured in the control simulations. On the other hand, for DRI-W6I and DRI-W6Y we see in Figure 4b,c a minimum in the relative RMSF for the K80-S100 segment indicating that presence of the two peptides does not increase the flexibility of that region over that of the control, i.e., does not weaken the interactions between the polar cavity (strand G) and the adjacent strand H. Consistent with our hypothesis are RMSD values in Figure 2 for the two peptides also lower than for the control, and the final conformations in the inset do not show the loosening of fibril structure seen in the 400 K trajectory or in the DRI-K6V simulations.

The above observations are quantified by comparing the time evolution of the radius of gyration (*R*_g_) and the solvent-accessible surface area (SASA). We list in Table 2 for these quantities the difference between the averages over three trajectories and the corresponding start values. The absolute values can be found in the Supporting Information (Tables ST3 and ST4).

**Table 2:** Difference between the Averages of Various Quantities, Taken over the Last 200 ns of Three Independent Trajectories, and the Corresponding Start Values as Measured in Simulations of the TTR Fibril in the Presence and Absence of the Three DRI Peptides

	Control	DRI-K6V	DRI-W6I	DRI-W6Y
whole fibril				
*R*_g_ (Å)	0.02 (0.02)	0.07 (0.01)	0.04 (0.02)	0.01 (0.01)
total SASA (Å^2^)	407 (512)	1794 (169)	928 (476)	699 (267)
hydrophobic SASA (Å^2^)	692 (278)	966 (152)	261 (382)	158 (260)
hydrophilic SASA (Å^2^)	–285 (294)	828 (120)	666 (126)	541 (116)
N-terminus				
*R*_g_ (nm)	0.03 (0)	0.04 (0)	0.04 (0.01)	0.03 (0.01)
total SASA (Å^2^)	–894 (279)	–522 (135)	–1047 (140)	–778 (268)
hydrophobic SASA (Å^2^)	–219 (121)	–384 (179)	–566 (109)	–534 (265)
hydrophilic SASA (Å^2^)	–676 (158)	–137 (57)	–481 (31)	–243 (42)
C-terminus				
*R*_g_ (nm)	0.05 (0.02)	0.09 (0.01)	0.07 (0.02)	0.04 (0.01)
total SASA (Å^2^)	1301 (417)	2315 (184)	1975 (485)	1476 (207)
hydrophobic SASA (Å^2^)	911 (287)	1350 (54)	828 (408)	692 (51)
hydrophilic SASA (Å^2^)	391 (174)	965 (136)	1148 (110)	785 (156)
cavity				
*R*_g_ (nm)	0.1 (0.09)	0.35 (0.20)	0.16 (0.07)	0.09 (0.05)
total SASA (Å^2^)	1032 (485)	1852 (290)	1688 (454)	1291 (183)
hydrophobic SASA (Å^2^)	897 (301)	1125 (195)	981 (307)	871 (80)
hydrophilic SASA (Å^2^)	153 (214)	727 (95)	705 (163)	420 (121)
residues K80-S100				
*R*_g_ (nm)	0.03 (0.01)	0.03 (0.02)	0.01 (0.01)	0.01 (0.01)
total SASA (Å^2^)	457 (120)	511 (265)	455 (288)	337 (34)
hydrophobic SASA (Å^2^)	284 (88)	440 (197)	288 (241)	125 (20)
hydrophilic SASA (Å^2^)	173 (37)	71 (69)	167 (48)	211 (16)

The radius of gyration (*R*_g_) is a measure of the compactness of conformations and increases for DRI-K6V and DRI-W6I while changing little in DRI-W6Y and the control. The change mainly results from the C-terminal segment (residues G57-E127) and especially the polar cavity. The loosening of the TTR fibril structure leads for DRI-K6V and DRI-W6I also to a pronounced increase in solvent-accessible surface area (SASA), resulting again mostly from the C-terminal segment and especially the polar cavity. This increase in SASA mainly results from exposure of hydrophilic residues, but in the case of DRI-K6V, also from strong exposure of hydrophobic residues.

The changes in *R*_g_ and SASA are correlated with a loss of interchain contacts in the C-terminal segment and again especially pronounced for DRI-K6V and its polar cavity. We list in Table 3 the difference between the averages of native interchain contacts, that is, contacts that exist also in the start conformation, over three trajectories and the corresponding start values. The absolute values can be found in the Supporting Information Table ST5. When counting only native contacts, we find in the control an overall loss of 270 (7) contacts, only slightly larger losses of 304 (15) for DRI-W6I and 283 (12) for DRI-W6Y, but with 360 (7), a much larger loss for DRI-K6V. The loss results mainly from stalling contacts between the layers of the fibril and less from the packing contacts between the N-terminal and C-terminal fragments. For instance, in the presence of DRI-K6V, 79 more stagging contacts are lost than in the control, but only nine more packing contacts. The loss in stagging contacts is mostly from the cavity, where on average 30 more contacts are lost in the DRI-K6 V simulation than in the control, and from the segment of residues K80-S100, where in the DRI-K6V simulations 10 more contacts are lost than in the control, while for this segment, 11 contacts less are lost for DRI-W6I and 21 less for DRI-W6Y.

**Table 3:** Difference between the Averages of Contacts, Taken over the Last 200 ns of Three Independent Trajectories, and the Corresponding Start Values as Measured in Simulations of the TTR fibril in the Presence and Absence of the Three DRI Peptides^a^

		control	DRI-K6V	DRI-W6I	DRI-W6Y
whole fibril	interchain contacts	–270 (7)	–360 (7)	–304 (15)	–283 (12)
	packing contacts	–35 (5)	–46 (2)	–45 (1)	–34 (6)
	stagging contacts	–235 (3)	–314 (8)	–259 (11)	–249 (12)
N-terminus	stagging contacts	–50 (2)	–65 (10)	–56 (7)	–60 (5)
C-terminus	stagging contacts	–177 (4)	–232 (9)	–179 (9)	–180 (9)
cavity	stagging contacts	–92 (6)	–122 (8)	–98 (4)	–109 (7)
residues K80-S100	stagging contacts	–45 (13)	–55 (13)	–34 (12)	–24 (3)

a Only native contacts, that is contacts already found at the start, are considered.

Visual inspection of the final conformations shows that the changes to the fibril conformations are mainly in the outer layers, which, especially in the unphysical 400 K simulation, but also in the presence of DRI-K6V, get partially separated from the rest of the fibril. For this reason, we have also measured the stacking contacts between chain 1 (the bottom chain) and chain 2 and between chain 4 and chain 5 (the top chain), comparing again simulations where one of the DRI peptides is present with the control simulations. Focusing again on native contacts, we have listed in Table 4 the differences between averages over the last 200 ns with the corresponding start values. Absolute values are listed in the supplemental Table ST6. Quantifying our visual impression of a partial separation of the outer layers, we find that in the control 126 (7) such stagging contacts between the outer chains and the core fibril are lost, but 163 (4) in the presence of DRI-K6 V and only 135 (14) in the presence of DRI-W6I and 127 (12) in the presence of DRI-W6Y. While all parts of the TTR fibril lose such stacking contacts, the relative loss is especially strong for the segment K80-S100, where in the control only 6 (about 6%) such contacts are lost, but 12 (12%) in the presence of DRI-K6V, 9 (9%) in the presence of DRI-W6I, and 4 (4%) in the presence of DRI-W6Y. Note that these percentages are averages over only three independent trajectories of the time averages taken over the last 200 ns in each trajectory. Differences between the time averages of the trajectories are much larger than the standard deviations associated with each time average; see for example, supplemental Table ST7. While the resulting standard deviations for the percentages seen in Table 4 are large, our data indicate that interaction of DRI-K6V with the TTR fibril leads to a partial separation of the outer chains for the segment K80-S100, while this is not the case for the other two peptides.

**Table 4:** Native Stacking Contacts between the Outer Chains and the Core of the TTR Fibril (i.e., between Chain 1 and Chain 2, and between Chain 4 and Chain 5)^a^

	control	DRI-K6 V	DRI-W6I	DRI-W6Y
whole fibril	–126 (7)	–163 (4)	–135 (14)	–127 (12)
N-terminus	–23 (3)	–34 (9)	–25 (2)	–29 (5)
C-terminus	–98 (3)	–122 (10)	–100 (14)	–93 (10)
cavity	–52 (5)	–63 (6)	–51 (7)	–58 (5)
residues K80-S100	–6 (4)	–12 (13)	–9 (9)	–4 (4)

a Shown are the differences between the averages taken over the last 200 ns of three independent trajectories and the corresponding start values as measured in simulations of the TTR fibril in the presence and absence of the three DRI peptides.

## Conclusions

Damage to the heart and other organs in elderly patients is often caused by transthyretin (TTR) amyloid deposits. The lack of treatment options targeting these deposits directly is caused in part by the lack of a suitable animal model for this TTR amyloidosis, making *in vivo* experiments difficult and costly. Hence, detailed knowledge of the mechanism by which inhibitors could dissolve the TTR fibril is needed for optimizing the potency and selectivity of potential drug candidates prior to experimental validation. This medical question motivated us to look into the possibility of modulating the stability of TTR fibrils with short peptides. Comparing molecular dynamics simulations at an unphysiological high temperature of 400 K with those at the physiological temperature of 310 K, we find indications that disruptions of the fibril structure start with the segment of residues K80-S100, which connects the polar cavity with the adjacent strand H. Using virtual screening, we designed three peptides and evaluated, in molecular dynamics (MD) simulations at physiological temperatures, their ability to destabilize TTR amyloid fibrils. In order to ease future experimental validation, we designed the peptides from D-amino acids, as these do not suffer from the short in vivo lifetimes of regular peptide drugs.

In only one case, for DRI-K6V, we see evidence of increased structural disorder and destabilization of the TTR fibril. While limited by the small number of trajectories and large fluctuations between them, our data indicate that this process begins with partial segregation of the outer chains, starting again with the segment of residues K80-S100, where it appears that double as many contacts are lost as in the control. Subsequent losses of stagging contacts in the outer layers in the polar cavity lead to a further weakening that may indicate an avenue for dissolving the TTR fibril. Such weakening of outer β-strand contacts and nearby loops, followed by disruption of interchain networks within the fibril core, has also been suggested in other recent work on TTR fibril destabilization. For instance, it was proposed that the small molecule epigallocatechin gallate (EGCG) destabilizes the mutant V30M TTR fibrils by binding to boundary β-strands and promoting their dissociation.^72^ Additional MD studies suggested that EGCG lowers β-strand content in TTR fibrils, specifically in the cavity region, by destabilizing the L58-I84 contact network at the cavity entrance, while EGC (lacking a gallic acid ester group) binds within the cavity and weakens salt bridges that help maintain its structure.^73^ Unlike this earlier work, our results emphasize the role of the segment K80-S100 as a potential target for modulating TTR fibril stability.

A limitation of our study is that we did not explore potential differences in the efficiency of the peptides to disrupt TTR fibrils between wild type and the mutants causing hereditary forms of TTR amyloidosis as, for instance, the V30M mutation considered in the computational study^72^ mentioned above. Any medical application of DRI-K6V would have to consider also its binding to competing proteins in the heart and other organs. Additionally, future studies would need to determine whether the peptide–fibril interactions identified here are maintained at substantially lower peptide concentrations that are relevant for potential pharmacological applications. We hope that by describing a mechanism for disrupting TTR fibrils with DRI peptides, we have built a foundation for targeting these questions in future work.

## Supplementary Material







## Data Availability

The data that support the findings of this study are available in the Supporting Information and are publicly accessible at https://github.com/ouhansmannlab/transthyretin_fibril.git

## Notes

Seven supplemental tables with statistics of the simulations and supporting data (PDF)
Start and final configurations of all trajectories in PDB format as text files in a compressed folder (ZIP)
Topology files and. itp files for each system are provided in a compressed folder (ZIP)

## Abbreviations

ATTR: transthyretin amyloidosis
ATTRwt: wild-type transthyretin amyloidosis
ATTRv: hereditary transthyretin amyloidosis
DRI: D-Retro-Inverso
FDA: U.S. Food and Drug Administration
*R*_g_: radius of gyration
RMSD: root-mean-square deviation
RMSF: root-mean-square fluctuation
SAA: serum amyloid A
SASA: solvent-accessible surface area
TTR: transthyretin
