# Supplementary material for: Modulating Transthyretin Fibril Stability with D‑Retro-Inverso Peptides
Source: J Chem Inf Model. 2026 Oct 27;66(21):14115–25. doi: 10.1021/acs.jcim.6c02525 (PMC13753513; doi:10.1021/acs.jcim.6c02525)
Supplement: Supplementary file 1 [file ci6c02525_si_001.pdf]

Supporting Information

Modulating Transthyretin Fibril Stability

with D-Retro Inverso Peptides

Lucy M. Coleman and Ulrich H. E. Hansmann*

Department of Chemistry & Biochemistry, University of Oklahoma, Norman, Oklahoma 73019, United States.

E-Mail: uhansmann@ou.edu

Supporting information for this article consists of 7 tables and 2 figures listed on the following pages and two separate files, (**TTR-fibril-coordinates.zip**) a compressed folder with the atomic coordinates of the start and final configurations (as ASCII files in the PDB format) of all trajectories and (**TTR-fibril-topology.zip**) a compressed folder with the topology files and ITP files (separated by chain) of all trajectories of the molecular dynamics simulations considered in this study. The folders contain also README files to help identifying the various files.

Table of Contents of Supporting Information

Tables:

ST1: Grouping System based on Properties of Amino Acids. S3

ST2: Top 10 peptide candidates ranked by Rosetta binding free energy estimate. ΔG values are reported in Rosetta Energy Units (REU) and represent a computational estimate of binding free energy. S4

ST3: Radius of gyration (Rg) measured over the last 200 ns in simulations. Averages over three trajectories for each system are shown, with standard deviations in parenthesis. S5

ST4: Solvent accessible surface area (SASA) measured over the last 200 ns in simulations. Averages over three trajectories for each system are shown, with standard deviations in parenthesis. S6

ST5: Native contacts measured over the last 200 ns in simulations. Averages over three trajectories for each system are shown, with standard deviations in parenthesis. S7

ST6: Native stagging contacts between the outer chains and the core of the TTR fibril (i.e. between chain 1 and chain 2, and between chain 4 and chain 5) measured over the last 200 ns in simulations. Averages over three trajectories for each system are shown, with standard deviations in parenthesis. S8

ST7: Native stagging contacts between the outer chains and the core of the TTR fibril (i.e. between chain 1 and chain 2, and between chain 4 and chain 5) in the region K80-S100. Shown are both time averages for each of the three trajectories for each system. The last row shows the mean of the three run averages, with the sample standard deviation taken across the three run averages. S9

Figures:

SF1: Structures of the recently in Ref. 59 proposed inhibitors docked to the TTR fibril: (a) TabF2 in cyan and (b) TabH2 in green. S10

SF2: Per-chain binding probability to the TTR fibril for the three considered peptides. S11

README for additional files in the compressed folder TTR-fibril-coordinates.zip (in separate file) S12

README for aditional files in the compressed folder TTR-fibril-topology.zip (in separate file) S14

Table ST1. Grouping System based on Properties of Amino Acids

**Nonpolar, Aliphatic**	**Polar, Uncharged**	**Negatively Charged**	**Aromatic**
Glycine (Gly, G)	Serine (Ser, S)	Aspartate (Asp, D)	Phenylalanine (Phe, F)
Alanine (Ala, A)	Threonine (Thr, T)	Glutamate (Glu, E)	Tyrosine (Tyr, Y)
Valine (Val, V)	Cysteine (Cys, C)		Tryptophan (Trp, W)
Proline (Pro, P)	Asparagine (Asn, N)	**Positively Charged**	
Leucine (Leu, L)	Glutamine (Gln, Q)	Lysine (Lys, K)	
Isoleucine (Ile, I)		Histidine (His, H)	
Methionine (Met, M)		Arginine (Arg, R)	

Table ST2. Top 10 peptide candidates ranked by Rosetta binding free energy estimate. ΔG values are reported in Rosetta Energy Units (REU) and represent a computational estimate of binding free energy.

Rank	Peptide	ΔG (REU)	Buried SA ($Å^2$)	Interface H-bonds	Unsatisfied H-bonds
1	DRI-KALGIV	-27.135	1239.247	4	3
2	DRI-GAAIGW	-27.051	1119.133	5	2
3	DRI-YTIGAL	-25.977	1357.379	8	4
4	DRI-VALHGF	-25.769	1305.09	6	13
5	DRI-STWIFE	-23.989	1251.187	3	12
6	DRI-WKALLI	-23.874	1438.996	3	17
7	DRI-LKVLDA	-23.752	1364.675	7	12
8	DRI-WTFNLY	-23.18	1268.806	6	6
9	DRI-GYFLNF	-22.969	1343.166	4	12
10	DRI-AVHLFK	-22.804	1383.584	3	5

Table ST3. Radius of gyration (nm) measured over the last 200 ns in simulations. Averages over three trajectories for each system are shown, with standard deviations in parenthesis.

	**Control**		**DRI-K6V**		**DRI-W6I**		**DRI-W6Y**	
	Initial	Last 200 ns	Initial	Last 200 ns	Initial	Last 200 ns	Initial	Last 200 ns
Whole Fibril	2.36	2.38 (0.02)	2.37	2.44 (0.01)	2.38	2.42 (0.02)	2.37	2.38 (0.01)
N-terminus	1.38	1.41 (0)	1.38	1.41 (0)	1.38	1.42 (0.01)	1.38	1.41 (0.01)
C-terminus	2.13	2.19 (0.02)	2.15	2.24 (0.01)	2.15	2.22 (0.02)	2.15	2.19 (0.01)
Cavity	1.22	1.32 (0.09)	1.23	1.6 (0.2)	1.41	1.57 (0.07)	1.41	1.50 (0.05)
Residues K80-S100	1.33	1.36 (0.01)	1.34	1.37 (0.02)	1.35	1.36 (0.01)	1.35	1.36 (0.01)

Table ST4. Solvent accessible surface area (SASA) (Å^2) measured over the last 200 ns. Averages over three trajectories for each system are shown, with standard deviations in parenthesis.

		Control		DRI-K6V		DRI-W6I		DRI-W6Y	
		Initial	Last 200 ns	Initial	Last 200 ns	Initial	Last 200 ns	Initial	Last 200 ns
Total	Whole Fibril	23108	23515 (512)	23034	24828 (169)	22876	23804 (476)	22750	23449 (267)
	N-terminus	9127	8233 (279)	8706	8184 (135)	8677	7630 (140)	8685	7907 (268)
	C-terminus	13981	15282 (417)	14328	16643 (184)	14199	16174 (485)	14066	15542 (207)
	Cavity	4257	5289 (485)	4425	6277 (290)	4442	6130 (454)	4305	5596 (183)
	Residues K80-S100	4421	4878 (120)	4717	5228 (265)	4706	5161 (288)	4610	4947 (34)
Hydrophobic	Whole Fibril	11330	12022 (278)	11778	12744 (152)	11765	12026 (382)	11774	11932 (260)
	N-terminus	4626	4407 (121)	4721	4337 (179)	4561	3995 (109)	4671	4137 (265)
	C-terminus	6704	7615 (287)	7056	8406 (54)	7204	8032 (408)	7104	7796 (51)
	Cavity	1713	2610 (301)	1946	3071 (195)	2115	3096 (307)	2003	2874 (80)
	Residues K80-S100	2084	2368 (88)	2211	2651 (197)	2228	2516 (241)	2220	2345 (20)
Hydrophilic	Whole Fibril	11778	11493 (294)	11256	12084 (120)	11111	11777 (126)	10976	11517 (116)
	N-terminus	4501	3825 (158)	3984	3847 (57)	4116	3635 (31)	4014	3771 (42)
	C-terminus	7277	7668 (174)	7272	8237 (136)	6995	8143 (110)	6962	7747 (156)
	Cavity	2526	2679 (214)	2479	3206 (95)	2328	3033 (163)	2302	2722 (121)
	Residues K80-S100	2337	2510 (37)	2506	2577 (69)	2478	2645 (48)	2390	2601 (16)

Table ST5. Native contacts measured over the last 200 ns in simulations. Averages over three trajectories for each system are shown, with standard deviations in parenthesis.

		Control		DRI-K6V		DRI-W6I		DRI-W6Y	
		Initial	Last 200 ns	Initial	Last 200 ns	Initial	Last 200 ns	Initial	Last 200 ns
Native Interchain Contacts	Whole Fibril	1190	920 (7)	1229	869 (7)	1199	895 (11)	1208	925 (17)
Native Packing Contacts		126	91 (5)	128	82 (2)	123	78 (1)	125	91 (6)
Native Stagging Contacts		1064	829 (3)	1101	787 (8)	1076	817 (11)	1083	834 (12)
Native Stagging Contacts	N-terminus	262	212 (2)	269	204 (10)	270	214 (7)	266	206 (5)
	C-terminus	747	570 (4)	775	543 (9)	749	570 (9)	765	585 (9)
	Cavity	263	171 (6)	280	158 (8)	270	172 (4)	286	177 (7)
	Residues K80-S100	194	149 (13)	195	140 (13)	188	154 (12)	192	168 (3)

Table ST6. Native stagging contacts between the outer chains and the core of the TTR fibril (i.e. between chain 1 and chain 2, and between chain 4 and chain 5) measured over the last 200 ns in simulations. Averages over three trajectories for each system are shown, with standard deviations in parenthesis.

	**Control**		**DRI-K6V**		**DRI-W6I**		**DRI-W6Y**	
	Initial	Last 200 ns	Initial	Last 200 ns	Initial	Last 200 ns	Initial	Last 200 ns
Whole Fibril	530	404 (7)	543	380 (4)	538	403 (14)	530	403 (12)
N-terminus	125	102 (3)	132	98 (9)	132	107 (2)	128	99 (5)
C-terminus	377	279 (3)	383	261 (10)	381	281 (14)	376	283 (10)
Cavity	135	83 (5)	139	76 (6)	136	85 (7)	143	85 (5)
Residues K80-S100	96	90 (4)	97	85 (13)	95	86 (9)	94	90 (4)

Table ST7. Native stagging contacts between the outer chains and the core of the TTR fibril (i.e. between chain 1 and chain 2, and between chain 4 and chain 5) in the region K80-S100. Shown are the differences between the averages taken over the last 200 ns and the corresponding start values as measured in simulations of the TTR fibril in presence and absence of the three DRI-peptides. Individual runs show the standard deviation within that trajectory over the final 200 ns. The last row shows the mean of the three run averages, with the sample standard deviation taken across the three run averages.

	**Control**	**DRI-K6V**	**DRI-W6I**	**DRI-W6Y**
	Last 200 ns	Last 200 ns	Last 200 ns	Last 200 ns
Run 1	-9 (5)	-20 (5)	-4 (3)	-1 (3)
Run 2	-9 (3)	2 (3)	-19 (6)	-9 (3)
Run 3	-1 (3)	-20 (5)	-2 (3)	-1 (3)
Average of 3 Runs	-6 (5)	-12 (13)	-9 (9)	-4 (5)

Figure SF1. Structures of the recently in Ref. 59 proposed inhibitor docked to the TTR fibril: (a) TabF2 (in cyan) and (b) TabH2 (in green).

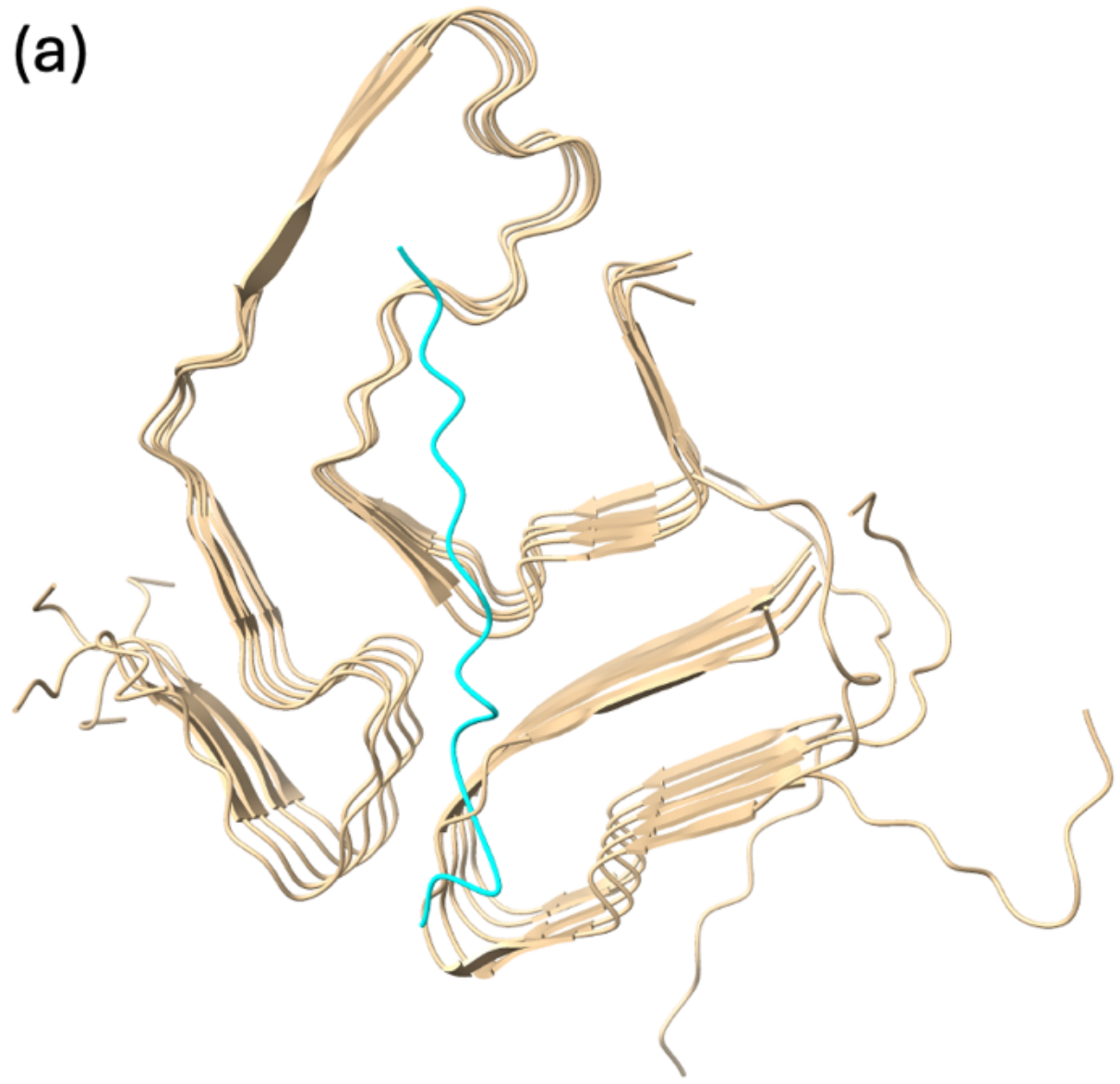

Saelices, L.; Chung, K.; Lee, J. H.; Cohn, W.; Whitelegge, J. P.; Benson, M. D.; Eisenberg, D. S. Amyloid seeding of transthyretin by ex vivo cardiac fibrils and its inhibition. *Proc Natl Acad Sci U S A* **2018**, *115* (29), E6741-E6750. DOI: 10.1073/pnas.1805131115.

Figure SF2. Per-chain binding probability to the TTR fibril for the three considered peptides over the last 200 ns.

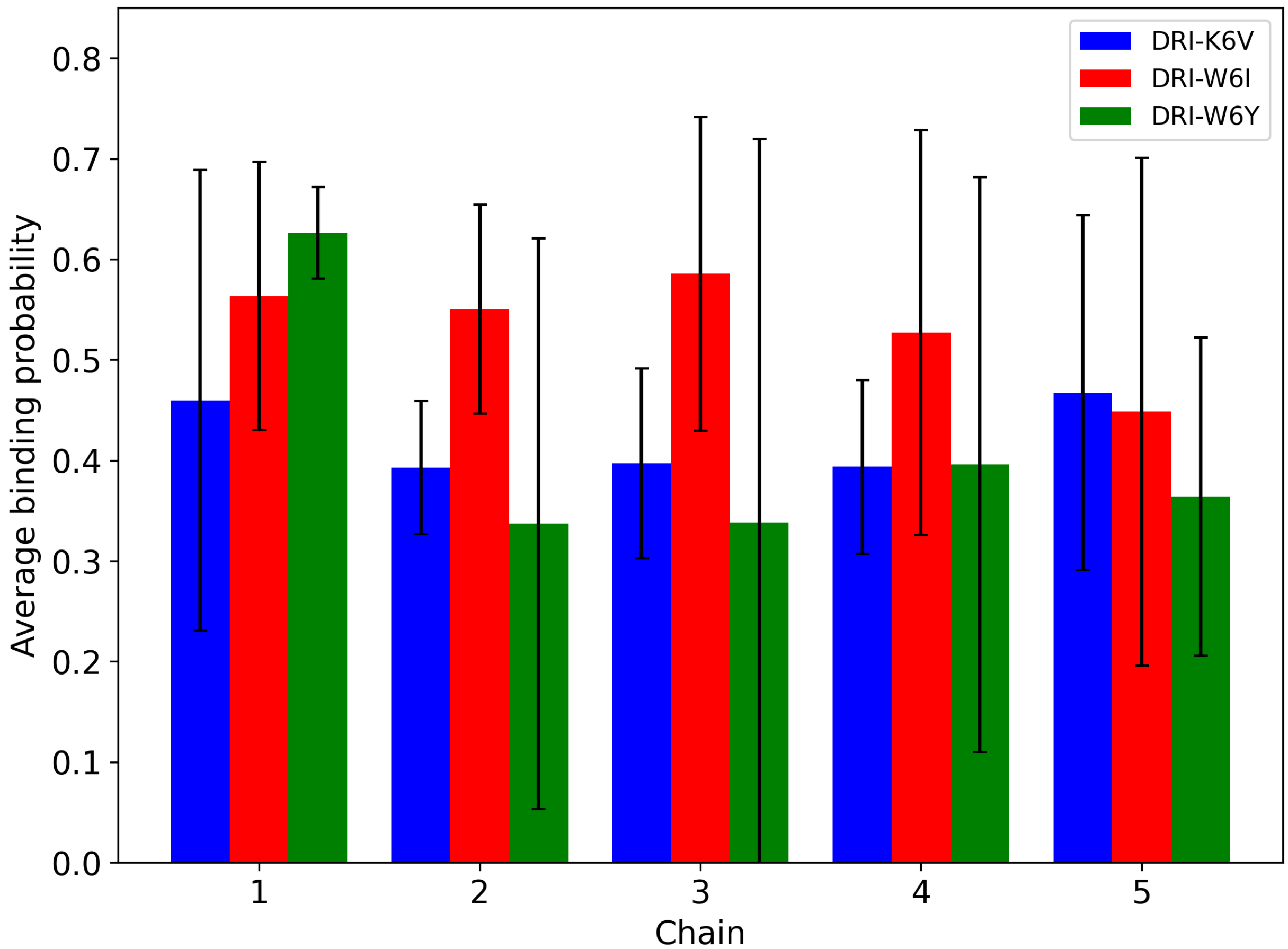

README for additional files in the compressed folder TTR-fibril-coordinates.zip (in separate file)

This directory contains (besides this README.txt) four folders (Control, K6V, W6Y, W6I) that contain files with the atomic coordinates (in PDB format) of TTR fibril structures discussed in the article. An additional folder (Parameter Files) contains mdp files of parameter settings for GROMACS simulations.

1) Control:

Atomic coordinates (in PDB format) of the start and final configurations of the control TTR fibril simulations:
control_initial_1 : Initial structure of TTR fibril replica 1
control_initial_2 : Initial structure of TTR fibril replica 2
control_initial_3 : Initial structure of TTR fibril replica 3
control_final_1 : Final structure of TTR fibril replica 1
control_final_2 : Final structure of TTR fibril replica 2
control_final_3 : Final structure of TTR fibril replica 3
control_400K_initial : Initial structure of TTR fibril in 400K control simulation
control_400K_final : Final structure of TTR fibril in 400K control simulation

2) K6V:

Atomic coordinates (in PDB format) of the start and final configurations of the TTR fibril simulations with K6V:
K6V_initial_1 : Initial structure of TTR fibril with K6V replica 1
K6V_initial_2 : Initial structure of TTR fibril with K6V replica 2
K6V_initial_3 : Initial structure of TTR fibril with K6V replica 3
K6V_final_1 : Final structure of TTR fibril with K6V replica 1
K6V_final_2 : Final structure of TTR fibril with K6V replica 2
K6V_final_3 : Final structure of TTR fibril with K6V replica 3

3) W6I:

Atomic coordinates (in PDB format) of the start and final configurations of the TTR fibril simulations with W6I:
W6I_initial_1 : Initial structure of TTR fibril with W6I replica 1
W6I_initial_2 : Initial structure of TTR fibril with W6I replica 2
W6I_initial_3 : Initial structure of TTR fibril with W6I replica 3
W6I_final_1 : Final structure of TTR fibril with W6I replica 1
W6I_final_2 : Final structure of TTR fibril with W6I replica 2
W6I_final_3 : Final structure of TTR fibril with W6I replica 3

4) W6Y:

Atomic coordinates (in PDB format) of the start and final configurations of the TTR fibril simulations with W6Y:

W6Y_initial_1 : Initial structure of TTR fibril with W6Y replica 1
W6Y_initial_2 : Initial structure of TTR fibril with W6Y replica 2
W6Y_initial_3 : Initial structure of TTR fibril with W6Y replica 3
W6Y_final_1 : Final structure of TTR fibril with W6Y replica 1
W6Y_final_2 : Final structure of TTR fibril with W6Y replica 2
W6Y_final_3 : Final structure of TTR fibril with W6Y replica 3

README for additional files in the compressed folder TTR-fibril-topology.zip (in separate file)

This directory contains (besides this README.txt) four folders (Control, K6V, W6I, W6Y) that contain topology files and ITP files (separated by chain) of the simulations discussed in the article.

1) Control:
topol.top : topology file describing the system
topol_Protein_chain_A.itp - topol_Protein_chain_J.itp : topology .itp files for individual TTR fibril chains
posre_Protein_chain_A.itp - posre_Protein_chain_J.itp : position restraint .itp files for individual TTR fibril chains

400K
topol.top : topology file describing the system
topol_Protein_chain_A.itp - topol_Protein_chain_J.itp : topology .itp files for individual TTR fibril chains
posre_Protein_chain_A.itp - posre_Protein_chain_J.itp : position restraint .itp files for individual TTR fibril chains

2) K6V:
topol.top : topology file describing the system
topol_Protein_chain_A.itp - topol_Protein_chain_J.itp : topology .itp files for individual TTR fibril chains
posre_Protein_chain_A.itp - posre_Protein_chain_J.itp : position restraint .itp files for individual TTR fibril chains

topol_Protein_chain_K.itp - topol_Protein_chain_O.itp : topology .itp files for individual K6V peptides
posre_Protein_chain_K.itp - posre_Protein_chain_O.itp : position restraint .itp files for individual K6V peptides

3) W6Y:
topol.top : topology file describing the system
topol_Protein_chain_A.itp - topol_Protein_chain_J.itp : topology .itp files for individual TTR fibril chains
posre_Protein_chain_A.itp - posre_Protein_chain_J.itp : position restraint .itp files for individual TTR fibril chains

topol_Protein_chain_K.itp - topol_Protein_chain_O.itp : topology .itp files for individual W6Y peptides
posre_Protein_chain_K.itp - posre_Protein_chain_O.itp : position restraint .itp files for individual W6Y peptides

4) W6I:
topol.top : topology file describing the system

topol_Protein_chain_A.itp - topol_Protein_chain_J.itp : topology .itp files for individual TTR fibril chains
posre_Protein_chain_A.itp - posre_Protein_chain_J.itp : position restraint .itp files for individual TTR fibril chains

topol_Protein_chain_K.itp - topol_Protein_chain_O.itp : topology .itp files for individual W6I peptides
posre_Protein_chain_K.itp - posre_Protein_chain_O.itp : position restraint .itp files for individual W6I peptides
